# 5-Methoxytryptophan enhances the sensitivity of sorafenib on the inhibition of proliferation and metastasis for lung cancer cells

**DOI:** 10.1186/s12885-024-11986-4

**Published:** 2024-02-22

**Authors:** Huang-Chi Chen, Chia-Yu Kuo, Yu Chang, Dong-Lin Tsai, Mei-Hsuan Lee, Jui-Ying Lee, Hui-Ming Lee, Yu-Chieh Su

**Affiliations:** 1 Division of Pulmonary and Critical Care Medicine, Department of Internal Medicine, Kaohsiung Municipal Siaogang Hospital, Kaohsiung, Taiwan; 2 Division of Pulmonary and Critical Care Medicine, Department of Internal Medicine, Kaohsiung Medical University Hospital, Kaohsiung Medical University, Kaohsiung, Taiwan; 3 Department of Obstetrics and Gynecology, E-Da Hospital, I-Shou University, Kaohsiung, Taiwan; 4 School of Medicine for International Students, College of Medicine, I-Shou University, Kaohsiung, Taiwan; 5 Department of Surgery, Kaohsiung Medical University Hospital, Kaohsiung Medical University, Kaohsiung, Taiwan; 6 Division of Chest Surgery, Department of Surgery, Kaohsiung Medical University Hospital, Kaohsiung Medical University, Kaohsiung, Taiwan; 7 Graduate Institute of Clinical Medicine, College of Medicine, Kaohsiung Medical University, Kaohsiung, Taiwan; 8 Division of General Surgery, Department of Surgery, E-Da Cancer Hospital, Kaohsiung, Taiwan; 9 School of Medicine, College of Medicine, I-Shou University, Kaohsiung, Taiwan; 10 Division of Hematology-Oncology, Department of Internal Medicine, E-Da Hospital, Kaohsiung, Taiwan

**Keywords:** Sorafenib, 5-methoxytryptophan, Lung cancer, Lung metastasis, Oncogenesis

## Abstract

**Background:**

Lung cancer is a leading cause of cancer-related mortality worldwide, and effective therapies are limited. Lung cancer is a leading cause of cancer-related mortality worldwide with limited effective therapy. Sorafenib is a multi-tyrosine kinase inhibitor frequently used to treat numerous types of malignant tumors. However, it has been demonstrated that sorafenib showed moderate antitumor activity and is associated with several side effects in lung cancer, which restricted its clinical application. This study aimed to examine the antitumor effect of the combination treatment of sorafenib and 5-methoxytryptophan (5-MTP) on cell growth and metastasis of Lewis lung carcinoma (LLC) cells.

**Method:**

The anticancer effect of the combination treatment of sorafenib and 5-MTP was determined through cytotoxicity assay and colony forming assays. The mechanism was elucidated using flow cytometry and western blotting. Wound healing and Transwell assays were conducted to evaluate the impact of the combination treatment on migration and invasion abilities. An in vivo model was employed to analyze the effect of the combination treatment on the tumorigenic ability of LLC cells.

**Result:**

Our results demonstrated that the sorafenib and 5-MTP combination synergistically reduced viability and proliferation compared to sorafenib or 5-MTP treatment alone. Reduction of cyclin D1 expression was observed in the sorafenib alone or combination treatments, leading to cell cycle arrest. Furthermore, the sorafenib-5-MTP combination significantly increased the inhibitory effect on migration and invasion of LLC cells compared to the single treatments. The combination also significantly downregulated vimentin and MMP9 levels, contributing to the inhibition of metastasis. The reduction of phosphorylated Akt and STAT3 expression may further contribute to the inhibitory effect on proliferation and metastasis. In vivo, the sorafenib-5-MTP combination further reduced tumor growth and metastasis compared to the treatment of sorafenib alone.

**Conclusions:**

In conclusion, our data indicate that 5-MTP sensitizes the antitumor activity of sorafenib in LLC cells in vitro and in vivo, suggesting that sorafenib-5-MTP has the potential to serve as a therapeutic option for patients with lung cancer.

**Supplementary Information:**

The online version contains supplementary material available at 10.1186/s12885-024-11986-4.

## Introduction

Lung cancer is one of the leading causes of cancer-related death worldwide, primarily attributed to late-stage diagnosis [1]. It is classified into two main histological groups: non-small cell lung cancer (NSCLC) and small cell lung cancer [2]. NSCLC accounts for 80–85% of all lung cancer cases and shows limited response to traditional cytotoxic chemotherapy [3, 4]. The 5-year survival rate of NSCLC is approximately 20–30%, significantly lower than that of other types of cancers, such as prostate, breast, and thyroid cancers [5]. Around 30% of NSCLC patients are known to have regional metastasis, and an additional 40% are diagnosed at the distant metastatic stage [6]. Therefore, the poor survival of NSCLC patients can be attributed to late diagnosis at advanced stages and the complex biology of the disease.

Tumor metastasis consists of a series of interrelated and sequential steps, involving invasion, migration, colonization, and angiogenesis in the metastatic spread of cancer cells from a primary tumor to distant sites [7]. Epithelial-mesenchymal transition (EMT) is considered a guiding principle underlying the initiation and progression of metastasis [8]. During EMT, the metastasizing cells proliferate rapidly and secrete proteolytic enzymes to degrade the blood vessel wall and extracellular matrix [9, 10]. The downregulation of epithelial proteins (such as E-cadherin) and the gain of mesenchymal proteins (such as vimentin) are hallmarks of this process, which signify poor prognosis and are associated with invasion and metastasis in multiple carcinomas [11]. Therefore, reducing metastasis can be one of strategies for lung cancer treatment.

A previous study has shown that sorafenib exhibits inhibitory activity, reducing proliferation, cellular metabolic activities, angiogenesis, and increasing apoptotic activity, which contributes to its anti-tumorigenic effects [12]. Sorafenib is a multi-tyrosine kinase inhibitor approved and used for the treatment of advanced renal carcinoma [13]. Over the years, sorafenib has also been approved for clinical use in treating unresectable hepatocellular carcinoma and metastatic differentiated thyroid cancer [14, 15]. By blocking various tyrosine and serine/threonine receptors, sorafenib inhibits the Ras/Raf/extracellular signaling-regulated kinase signaling cascade [16]. However, previous studies have shown that sorafenib does not improve the overall survival time of patients with advanced NSCLC [17]. Furthermore, sorafenib treatment is associated with several side effects, including rash, diarrhea, hand-foot syndrome, fatigue, and hypertension, which restrict its application for NSCLC patients [18]. Therefore, further investigation is warranted to explore methods for reducing the required treatment dose of sorafenib and increasing tumor sensitivity to sorafenib.

5-methoxytryptophan (5-MTP) is a 5-methoxyindole metabolite of tryptophan metabolism [19]. 5-MTP has been found to suppress inflammatory-induced cancer cell growth and migration while attenuating reactive oxygen species formation and excessive immune cell infiltration by controlling inflammation through the blockade of p38 and nuclear factor (NF)-κB activation [20, 21]. Additionally, the inhibitory effect of 5-MTP on EMT, cancer cell migration, and metastasis is achieved by inhibiting cyclooxygenase (COX)-2 expression [22]. Moreover, 5-MTP has been shown to reduce invasion and metastasis of oral cancer both in vitro and in vivo [23]. In a murine A549 xenograft tumor model, intraperitoneal injection of 5-MTP significantly reduced tumor growth by up to 50% compared to the vehicle control [24]. Therefore, 5-MTP holds potential as an antitumor agent and could find more applications as a supplemental treatment.

The anticancer mechanisms of sorafenib and 5-MTP have been demonstrated to target the same molecule, the signal transducer and activator of transcription 3 (STAT3) [25, 26], which plays a crucial role in tumor cell proliferation and survival [27]. Based on this, we hypothesized that the combination treatment of sorafenib and 5-MTP could have an additive effect in inhibiting cell growth and metastasis in Lewis lung carcinoma (LLC) cells, potentially reducing the adverse side effects of sorafenib treatment. In the present study, we found that 5-MTP sensitizes the anticancer activity of sorafenib, leading to the inhibition of cell proliferation and metastasis in LLC cells both in vitro and in vivo. Furthermore, the combined treatment of sorafenib and 5-MTP induced cell-cycle arrest and triggered apoptosis in LLC cells.

## Materials and methods

### Experimental animals

Six-week-old male wild-type C57BL/6 mice were purchased from BioLasco Taiwan Co. Ltd (Taipei City, Taiwan). All animal experiments were conducted in specific pathogen-free environments, in compliance with the Care and Use of Laboratory Animals guidelines. The mice were housed in controlled environments, with clean-air rooms, a 12-hour light-dark cycle, and 50–60% relative humidity. They received human care for 14 days to acclimatize before the start of the experiment. The mice were provided with a standard diet and water ad libitum.

### Cell culture and reagents

LLC cells were cultured in Dulbecco’s modified Eagle’s medium containing 10% fetal bovine serum, 1% non-essential amino acids, and 1% antibiotic-antimycotic, and maintained in a 5% CO2 atmosphere at 37 °C in an incubator. The LLC cells were purchased from the American Type Culture Collection (ATCC, Manassas, WV, USA). 5-MTP and sorafenib were purchased from Sigma-Aldrich (St. Louis, MO, USA).

### Cytotoxicity assay

LLC cells were seeded in 96-well plates at a density of 5 × 10^3^ cells per well and incubated for 24 h. Subsequently, the cells were treated with different concentrations of sorafenib (10, 20, 30, 40, 50, and 60 µM), or 5-MTP (0.5, 1, 1.5, and 2 mM), or a combination of sorafenib and 5-MTP at the indicated concentrations for an additional 24 h. Cell viability was assessed using the CellTiter-Glo 2.0 Cell Viability Assay (Promega, Madison, WI, USA) following the manufacturer’s instructions. Luminescence was measured using the Varioskan™ LUX multimode microplate reader (Thermo Scientific, USA).

### Colony forming assay

LLC cells were harvested from the culture dish by trypsinization and resuspended in DMEM supplemented with 10% FBS. The LLC cells were seeded at a density of 50 cells per well into a 24-well plate. Following an overnight incubation, the medium was removed, and the cells were treated with sorafenib and 5-MTP at different indicated concentrations for 24 h. Afterward, the medium was changed to DMEM supplemented with 10% FBS. The cells were then cultured for 7 days. After 7 days, the medium was removed, and the cells were washed with PBS three times before staining with crystal violet.

### Analysis of drug synergism

LLC cells were seeded at a density of 5 × 10^3^ cells per well in 96-well plates and incubated for 24 h. Subsequently, the cells were treated with sorafenib, 5-MTP, or a combination of sorafenib and 5-MTP at the indicated concentrations for another 24 h. The synergistic index was calculated as previously described. The Combination Index (CI) was used to analyze the interaction between the pure compounds when used as a mixture. In the equations, A and B represent the effects of each individual agent, and AB represents the effect of the combination. The CI was calculated as follows: CI = %AB / (%A × %B). Synergism was defined as CI < 1, additivity was defined as CI = 1, and antagonism was defined as CI > 1.

### Flow cytometry

After treatment LLC cells were harvested and washed twice in PBS. Cells were fixed with 70% ethanol and incubated with 1 mg/ml Ribonuclease A (Sigma-Aldrich, St. Louis, USA) for 30 min at 37 °C. Subsequently, cells were washed twice in PBS and stained with PI. DNA content was analyzed by flow cytometry on a FACSCalibur Cytometer. Data analysis was performed using FlowJo software.

### Western blotting

LLC cells were seeded in 6-well plates at a density of 1.5 × 10^6^ cells per well and treated with sorafenib and 5-MTP at the indicated concentrations for 24 h. After treatment, the cells were washed with phosphate-buffered saline (PBS) and lysed by adding ice-cold RIPA buffer supplemented with protease and phosphatase inhibitors (Thermo Scientific, USA). The cell lysates were then centrifuged, and the supernatant was separated on 10% SDS-polyacrylamide gels and transferred to PVDF membranes. Subsequently, blocking was performed using a blocking reagent (LEADGENE, Taiwan). Membranes were incubated with each primary antibody, diluted in TBS containing 0.1% Tween-20 and 5% non-fat dried milk. The membrane samples were probed with antibodies specific for anti-Vimentin (ABclonal; 1:3000, A2584), anti-MMP-9 (ABclonal; 1:3000, A0289), anti-N-cadherin (ABclonal; 1:1000, A3045), anti-E-cadherin (ABclonal; 1:1000, A3044), anti-p-STAT3 (ABclonal; 1:1000, AP0705), anti-p-Akt (ABclonal; 1:1000, AP0637), and anti-GAPDH (GeneTex; 1:10000, GTX100118). An enhanced chemiluminescence technique was used to detect the signal. The protein abundance of the samples was quantified using Image-J software following densitometric scanning.

### Cell migration assay

The cell migration assay was conducted using an in vitro wound-healing assay. LLC cells were seeded in a 6-well plate at a density of 1 × 10^5^ cells. After 24 h of incubation, a 20–200 µL micropipette tip was used to create a linear wound on the cellular monolayer, followed by careful washing with PBS to remove debris and excess medium. Subsequently, cells were treated with sorafenib and 5-MTP at the indicated concentrations for 24 h. The migration of cells towards the gap area was photographed, and the widths of the scratches were analyzed using ImageJ software.

### Cell invasion assay and Matrigel transwell assay

The cell invasion and Matrigel transwell assays were conducted using 6.5 mm and 8 μm transwell plates (Corning Life Sciences, Corning, NY, USA), respectively, pre-coated with Matrigel® basement membrane matrix (BD Biosciences, Franklin Lakes, NJ, USA). Briefly, LLC cells were seeded at a density of 1 × 10^5^ cells in 200 µL of 2% FBS medium into the top chamber, either on a polycarbonate membrane insert or a membrane insert coated with Matrigel. The bottom chamber contained 700 µL of complete culture medium, including 10% FBS, to attract invading cells. The top chambers were treated with sorafenib and 5-MTP at the indicated concentrations for 24 h. Afterward, the chambers and wells were washed twice with PBS, and the cells were fixed with 4% paraformaldehyde for 20 min. Both chambers and wells were washed with PBS and soaked with 100% methanol for 20 min. Before staining, the chambers and wells were rinsed twice with PBS and stained with 0.5% crystal violet for 20 min. Then, the crystal violet was removed, and the number of cells that invaded the membranes was counted. A comparison was made with the number of cells that passed through the membrane in the control chambers, and photographs were taken. The numbers of invading and migrating cells were analyzed using ImageJ software.

### Tumor model

C57BL/6 mice were intravenously injected with 5 × 10^5^ LLC cells suspended in 100 µL of MEM serum-free medium via the tail vein, using a 0.5 mL syringe with a 27G needle. The mice were randomly divided into three groups: Group 1 received saline; Group 2 received sorafenib (1 mg/kg); Group 3 received a combination of sorafenib (1 mg/kg) and 5-MTP (35 mg/kg). Seven days after the injection of tumor cells, the mice were administered either saline, sorafenib (1 mg/kg), or the combination of sorafenib and 5-MTP (35 mg/kg) three times a week by intraperitoneal injection. After 3 weeks of treatment, C57BL/6 mice were sacrificed by CO2 euthanasia, and lung tissue was collected and fixed in 10% formalin for H&E staining. The tumor area in the lung was quantified using Image-J software.

### Histopathology

All tissues were fixed in 10% formalin for 48 h. In brief, each tissue was embedded in paraffin, cut into 3 μm-thick sections on slides, and subjected to H&E staining to observe tumor growth under a photomicroscope.

### Statistical analysis

Statistical analysis was conducted using Prism 9.2 (GraphPad Software Inc., USA). The Student’s t-test was employed to compare the statistical significance of differences between the control and drug-treated groups. Statistical significance was determined at *P* < 0.05.

## Results

### Sorafenib and 5-MTP attenuated the cell viability and proliferation in LLC cells

**Fig. 1:**
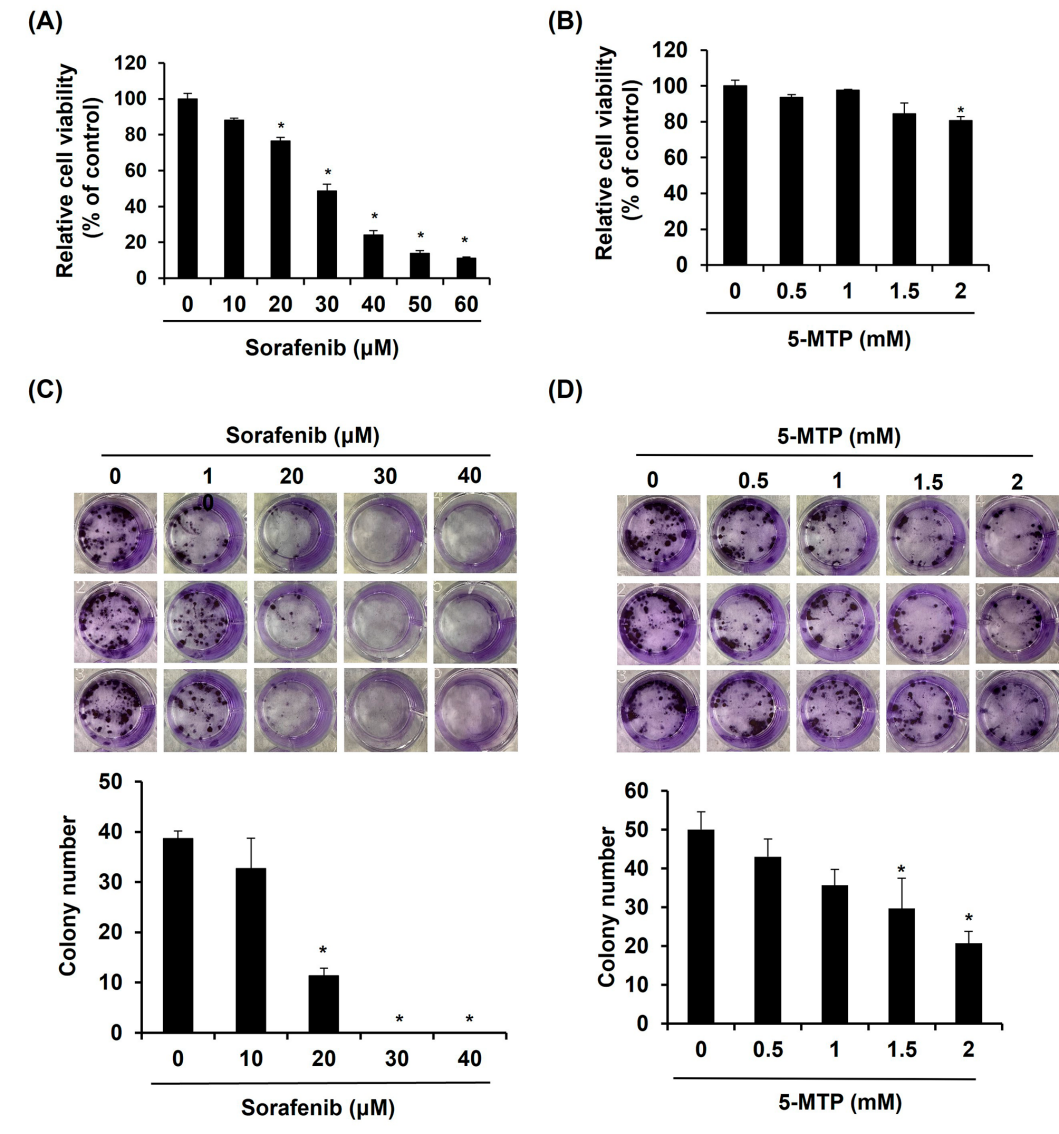
Reduction effect of sorafenib and 5-methoxytryptophan (5-MTP) on the cell viability and proliferation of Lewis lung cancer (LLC) cells. The viability of LLC cells was reduced by sorafenib (**A**) or 5-MTP (**B**) treatments in cytotoxicity assay. The proliferation of LLC cells was inhibited by sorafenib (**C**) or 5-MTP (**D**) treatments in colony forming assay. Asterisks indicate a significant difference in drug-treated cells at indicated concentrations singly compared to DMSO-treated cells. *P < 0.05

To investigate the anticancer activity of sorafenib and 5-MTP in lung cancer cells, LLC cells were treated with different doses of sorafenib or 5-MTP for 24 h. Cell viability was determined using a cell viability assay. The results showed that the presence of sorafenib at 10, 20, 30, 40, 50, and 60 µM dose-dependently reduced the cell viability of LLC after 24 h of treatment (Fig. 1A). The half maximal inhibitory concentration (IC50) of sorafenib in LLC cells was found to be 31.1 µM. Similarly, LLC cells treated with different concentrations of 5-MTP (0.5, 1, 1.5, and 2 mM) showed a significant reduction in cell viability, particularly at 2 mM of 5-MTP (Fig. 1B). However, since there was no clear dose-dependent reduction activity of 5-MTP, the IC50 of 5-MTP could not be accurately calculated. To further investigate the effect of sorafenib and 5-MTP on cell proliferation, LLC cells were treated with various concentrations of these compounds for 7 days, and the cell proliferation was determined using a colony forming assay. The results demonstrated that sorafenib dose-dependently reduced cell proliferation in LLC cells, and complete inhibition of proliferation was observed at 30 µM of sorafenib (Fig. 1C). The effect of 5-MTP on cell proliferation was consistent with the cell viability results, as proliferation was reduced at 2 mM of 5-MTP (Fig. 1D).

### Combination treatment of sorafenib and 5-MTP synergistically reduced the cell viability and proliferation in LLC cells

**Fig. 2:**
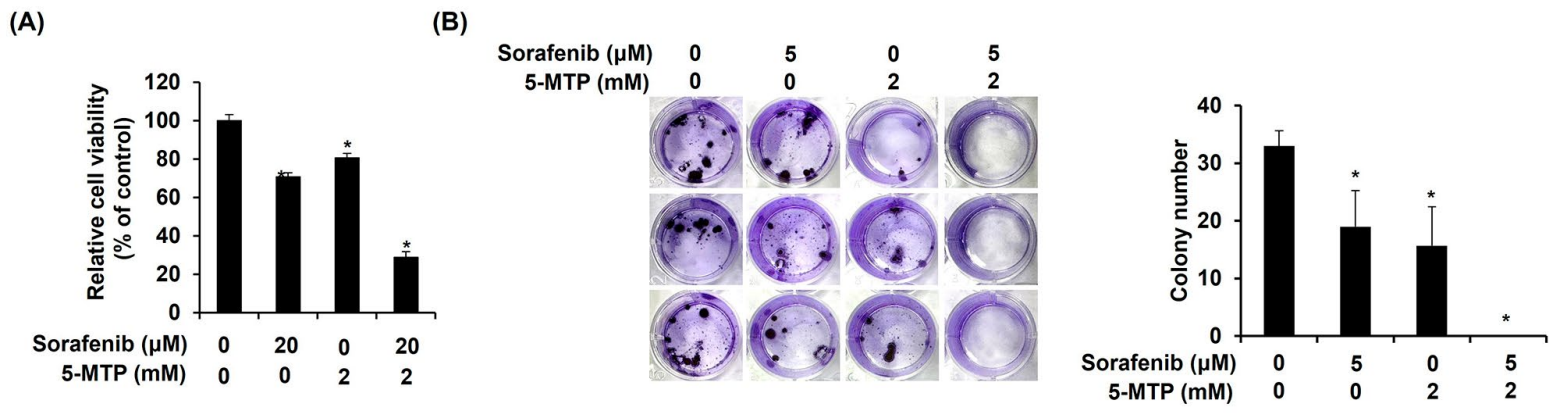
Synergistic inhibitory effect of the combination treatment of sorafenib and 5-MTP on the cell viability and proliferation of LLC cells. (**A**) Combination treatment showed a synergistic inhibitory effect on the growth of LLC cells compared to the single treatments in cytotoxicity assay. (**B**) The combination treatment induced the inhibition effect on cell proliferation. Asterisks indicate a significant difference in drug-treated cells at indicated concentrations singly compared to DMSO-treated cells. *P < 0.05

The effect of the combination treatment of sorafenib and 5-MTP on the growth of LLC cells was evaluated using a cell viability assay. LLC cells were treated with sorafenib and 5-MTP at combination concentrations of 20 µM and 2 mM or 30 µM and 2 mM for 24 h. The effect of the combination treatments was assessed using the coefficient of drug interaction (CDI) values, as described in the Materials and Methods section. The results showed that the CDI values for the combination treatments at the indicated concentrations were 0.50 and 0.46, respectively, suggesting a synergistic inhibitory effect in LLC cells (Fig. 2A). To further investigate whether 5-MTP sensitized the inhibitory effect of sorafenib on cell proliferation, LLC cells were treated with sorafenib and 5-MTP at concentrations of 5 µM and 2 mM for 7 days. The results showed that the combination treatment had a greater reduction in cell proliferation compared to the treatment with a single compound (Fig. 2B).

### Single and combination treatment of sorafenib and 5-MTP modulates cell cycle in

**Fig. 3:**
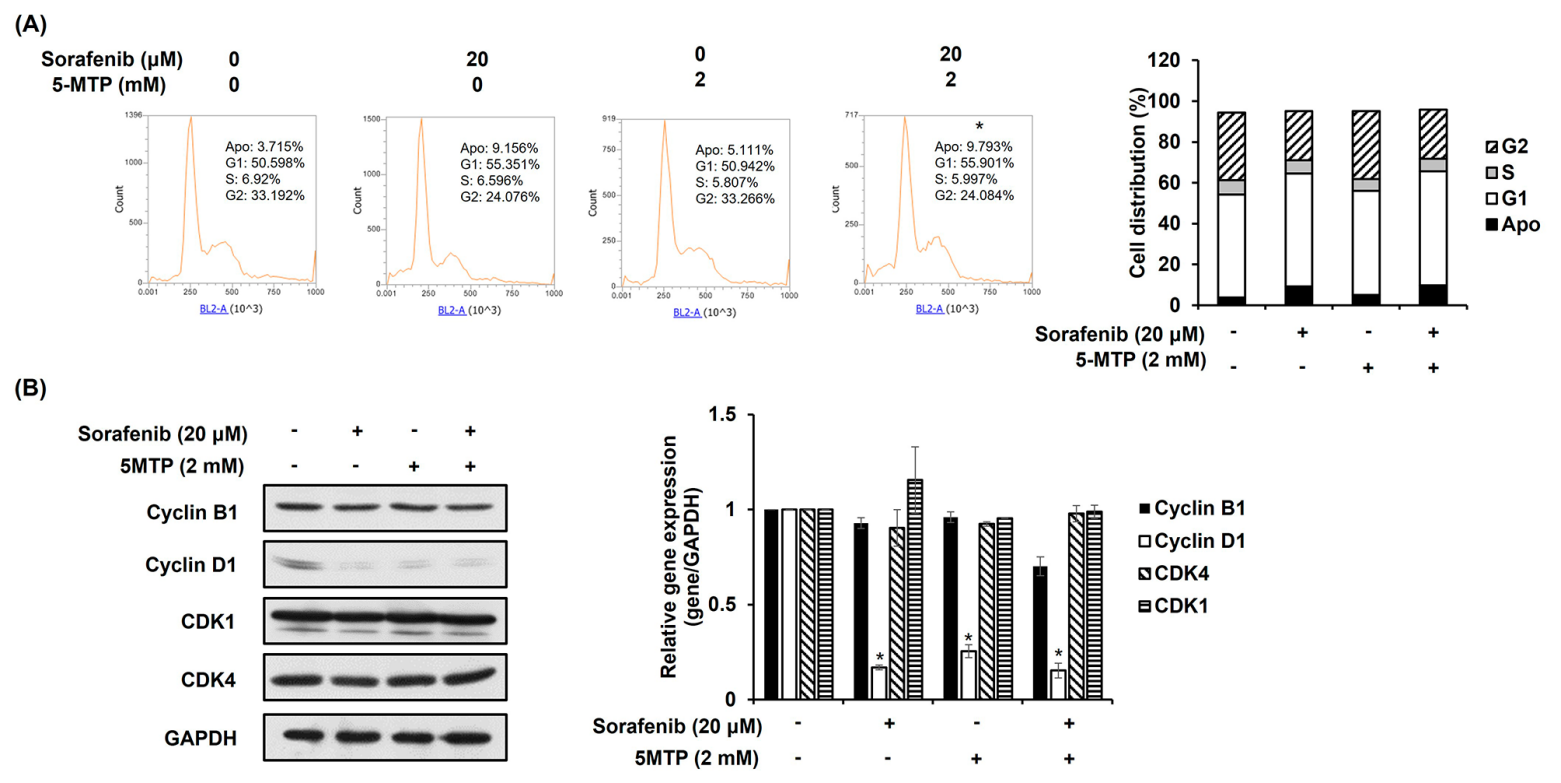
Induction of cell cycle arrest by sorafenib or combination treatment of sorafenib and 5-MTP in LLC cells. (**A**) Sorafenib and combination treatment induced cell cycle arrest, as observed by DNA staining with Propidium iodide and flow cytometry in LLC cells. (**B**) Cell cycle arrest was confirmed by western blot analysis 24 h after Sorafenib, 5-MTP, or combination treatment in LLC cells. Membranes were incubated with the indicated antibodies against cell cycle regulator proteins. GAPDH was used as a control for equal loading. *P < 0.05

As the combination treatment of sorafenib and 5-MTP decreased cell viability and proliferation in LLC cells, the cell cycle was investigated to understand how the inhibition was associated. When compared to the control and the single treatments with 5-MTP, sorafenib alone increased the percentage of cells in the G1 phase (Fig. 3A). The combination of sorafenib and 5-MTP at 20 µM and 2 mM showed a similar percentage of cells in G1, both higher than the percentage observed with 5-MTP treatment alone (Fig. 3A). This suggests that the combination treatment also induced G1 phase arrest, similar to sorafenib alone. To confirm the result of cell cycle arrest, the expression of cyclin B1, cyclin D1, CDK4, and CDK1 were determined by western blotting. The results showed that both sorafenib and the combination treatment reduced the expression of cyclin D1 (Fig. 3B), which is consistent with the induction of cell cycle arrest by these treatments. Overall, these results indicate that the combination treatment of sorafenib and 5-MTP induces cell cycle arrest, potentially contributing to the observed reduction in cell viability and proliferation in LLC cells.

### Combination treatment of sorafenib and 5-MTP increased the reduction effect on migration and invasion in lung cancer cells

**Fig. 4:**
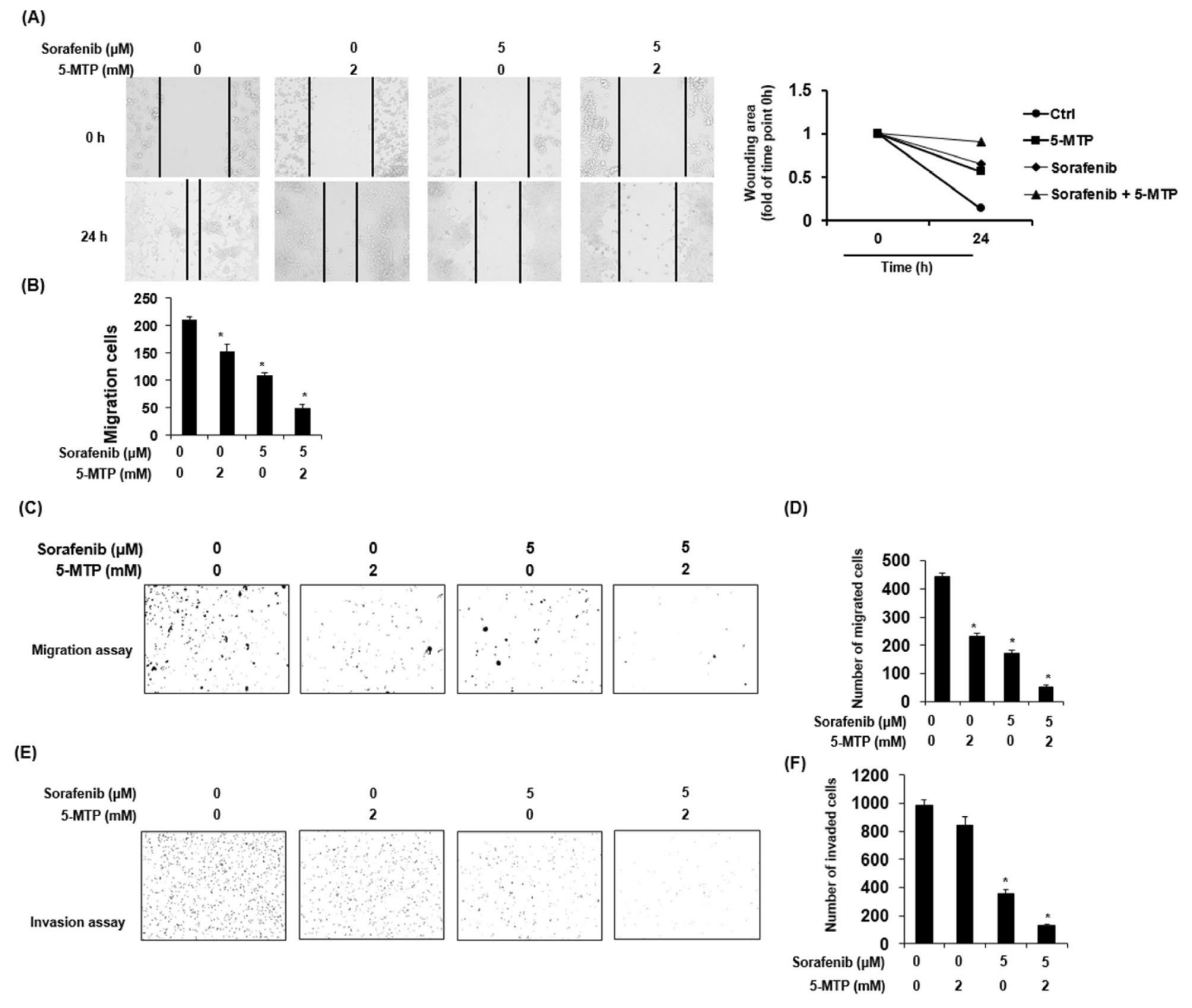
Enhanced inhibitory effect of sorafenib by 5-MTP on cell migration and invasion measured by wound-healing and transwell assay. (**A**) Representative bright-field images show that 5-MTP enhanced the reduction effect of sorafenib on migration compared to single compound treatment in wound-healing assay. (**B**) 5-MTP enhanced the reduction effect of sorafenib on the number of migration cells. (**C** and **E**) Representative bright-field images showed the effect of 5-MTP and sorafenib on cell migration and invasion in a transwell assay. (**D** and **F**) Statistic results showed that 5-MTP enhanced the inhibitory effect on cell migration and invasion. Asterisks indicate a significant difference in drug-treated cells at indicated concentrations singly compared to DMSO-treated cells. *P < 0.05

To investigate whether sorafenib, 5-MTP, or the combination of sorafenib and 5-MTP could reduce the mobility of lung cancer cells, wound-healing and transwell assays were performed. LLC cells were treated with sorafenib, 5-MTP, or the combination treatment at the indicated concentrations for 24 h. The results of the wound-healing assay demonstrated that the combination treatment of sorafenib and 5-MTP increased the reduction effect of the wound area compared to the single-agent treatments (Fig. 4A). Moreover, the combination treatment significantly attenuated the number of migrating cells compared to the control in the wound-healing assay (Fig. 4B). The inhibitory effect of the combination treatment on cell migration was further confirmed using the transwell migration assay, where the reduction in LLC migration was greater in the combination treatment group compared to the single-agent treatments (Fig. 4C and D). Additionally, the effect of sorafenib, 5-MTP, and the combination treatment on cell invasion was investigated. The results showed that the combination treatment notably enhanced the inhibition effect on the number of invasive lung cancer cells compared to the single-agent treatments (Fig. 4E and F). In summary, the combination treatment of sorafenib and 5-MTP demonstrated increased effectiveness in reducing cell mobility and invasion of lung cancer cells compared to the individual treatments, as evidenced by the wound-healing, transwell migration, and invasion assays.

### Combination treatment of sorafenib and 5-MTP decreased cell migration and invasion via mediating the expression of vimentin, matrix metalloproteinase-9 (MMP9), and E-cadherin

**Fig. 5:**
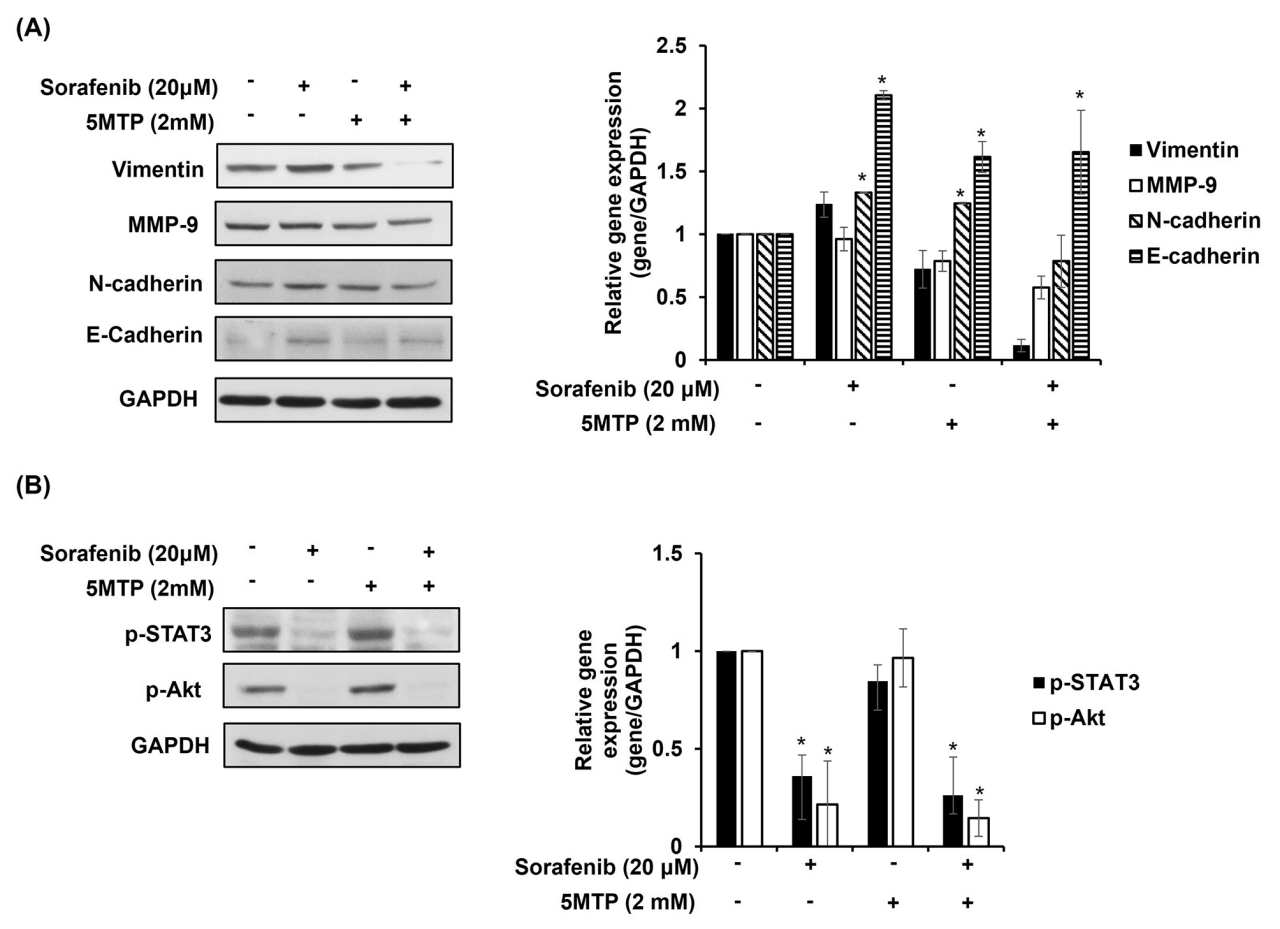
The effect of combination treatment of 5-TMP and sorafenib on the expression of EMT markers by mediating the expression of phosphorylated STAT3 and Akt. (**A**) Combination treatment of 5-MTP and sorafenib reduced the expression of vimentin and MMP9 and increased the E-cadherin level. (**B**) The level of phosphorylated STAT3 and Akt showed in western blotting. Asterisks indicate significant difference in drug-treated cells at indicated concentrations singly in comparison with DMSO-treated cells. *P < 0.05

The expression of several EMT markers was investigated in the drugs-treated cells and control cells to dissect the molecular mechanism of sorafenib and 5-MTP mediated cell migration and invasion in lung cancer cells. LLC cells were treated with sorafenib, 5-MTP, or the combination treatment at the indicated concentrations for 24 h, and the cell lysate was subjected to western blotting. The results showed a downregulation of vimentin and MMP9 expression and an increase in E-cadherin expression in sorafenib and 5-MTP-treated LLC cells (Fig. 5A). A previous study has shown that the Akt and STAT3 signaling pathways are involved in the EMT process in lung cancer cells [28].. LLC cells were treated with sorafenib, 5-MTP, or the combination treatment at the indicated concentrations for 24 h, and the levels of phosphorylated-Akt and -STAT3 were investigated by western blotting. As shown in Fig. 5B, the results demonstrated that sorafenib reduced the expression of phosphorylated-STAT3 and phosphorylated-Akt in LLC cells. When the combination treatment of sorafenib and 5-MTP was applied, it result in a more reduction in phosphorylated-STAT3 expression (Fig. 5B).

### Combination treatment of sorafenib and 5-MTP enhanced the reduction effect on tumor growth and metastasis in LLC-bearing mice

**Fig. 6:**
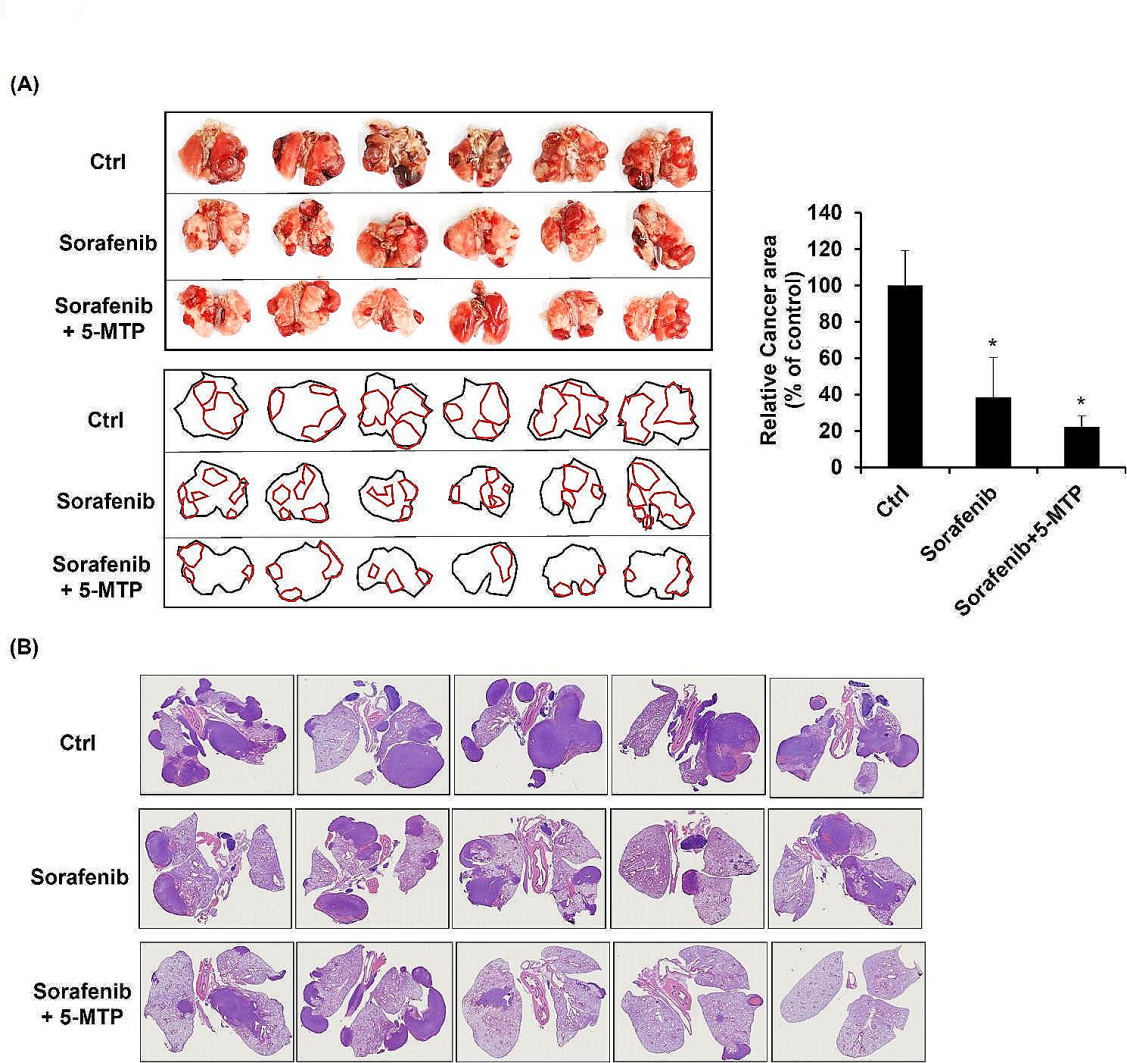
The inhibitory effect of sorafenib and combination treatment of sorafenib and 5-MTP on lung colony formation in vivo. (A) Morphological observation and digital outlines of representative tongues of the DMSO- or drug-treated group in the LLC mouse model. (B) hematoxylin–eosin staining of lung cancer with DMSO or drug treatment. *P < 0.05

The effect of the combination treatment on tumor growth in vivo was assessed using orthotopic LLC allografts in syngeneic C57BL/6 mice. To establish the LLC-bearing mice, LLC cells were injected via the tail vein. Seven days after cancer cell injection, the mice were treated with sorafenib, 5-MTP, or the combination of sorafenib and 5-MTP intraperitoneally three times a week for three weeks. Sorafenib treatment attenuated tumor growth in the lung tissue compared to the tumor control group. The combination treatment showed even more promising results, with 5-MTP enhancing the reduction effect of sorafenib on tumor growth both macroscopically (Fig. 6A) and microscopically (Fig. 6B). Histologic examination of tumors in H&E-stained lung sections revealed smaller tumor sizes and fewer LLC tumor nodules in the mouse lung tissue after treatment with the combination of sorafenib and 5-MTP (Fig. 6B). In conclusion, the combination treatment of sorafenib and 5-MTP demonstrated enhanced efficacy in reducing tumor growth in an in vivo model of lung cancer, as evidenced by both macroscopic and microscopic observations.

## Discussion

The present study demonstrated that 5-MTP enhances the inhibitory effect of sorafenib on cell viability, migration, and invasion compared to the treatment with sorafenib or 5-MTP alone in LLC cells. These findings suggest that 5-MTP effectively increases the sensitivity of lung cancer cells to sorafenib, potentially leading to a reduced dose requirement for sorafenib treatment in lung cancer. Further experiments revealed that the PI3K-AKT and STAT-3 signaling pathways are involved in the 5-MTP-induced sensitivity of cancer cells to sorafenib. In the LLC mouse model, 5-MTP further enhanced the inhibitory effect of sorafenib on the growth and metastasis of lung cancer cells. The data from both in vitro and in vivo studies demonstrate the success of the combination treatment of 5-MTP and sorafenib for lung cancer cells, suggesting that 5-MTP may have potential as a supplement to sorafenib for patients with lung cancer. Metastasis is the primary cause of death in cancer patients, especially in lung and breast cancers. Therefore, strategies that can effectively target and inhibit metastasis, such as the combination treatment of 5-MTP and sorafenib, hold great promise in improving the treatment outcomes for patients with these types of cancer [29]. EMT plays a critical role in tumor metastasis, which starts with primary tumor cell invasion. Endothelial cells exhibiting mesenchymal phenotype by loss of cell-to-cell adhesion that increases cell motility is typical phenomenon of EMT [29, 30]. Previous studies showed that 5-MTP reduces lung cancer A549 cell migration and invasion and attenuates A549 cancer growth by inhibiting COX-2 expression [31]. Furthermore, fibroblasts release 5-MTP to reduce cell EMT, migration, invasion, and metastasis [24]. In this study, 5-MTP significantly reduced lung cancer cell migration but did not inhibit invasion of the cells. Notably, the combination treatment of sorafenib and 5-MTP addictive reduced the invasion of lung cancer cell compared to the treatment of sorafenib alone. Typically, EMT includes the up-regulation of vimentin, N-cadherin, MMP9, and fibronectin expression, and the downregulation of E-cadherin expression [32]. The present study investigated the expression levels of vimentin, N-cadherin, E-cadherin, and MMP9, four important proteins involved in the EMT process, in LLC cells treated with sorafenib, 5-MTP, or both. Among these proteins, vimentin and MMP9 were reduced by the combination treatment of sorafenib and 5-MTP. Additionally, the combination treatment induced the level of E-cadherin. These results may explain the enhanced inhibitory effect of the combination treatment on cell migration and metastasis in LLC cells when compared to the treatment with sorafenib or 5-MTP alone. Interestingly, sorafenib alone elevated the expression of E-cadherin, while 5-MTP only slightly induced its level. Considering that EMT is crucial for initiating the process of tumor metastasis cascade, vimentin and MMP9 have garnered significant attention as essential markers for EMT [33]. Increased expression of vimentin and MMP9 can be found in various epithelial cancers, including lung, colorectal [34], and breast cancer [35, 36]. A previous study demonstrated that vimentin is required for lung cancer metastasis [37]. Furthermore, the suppression of MMP-9 expression inhibits the invasiveness and metastasis of tumor cells, reduces angiogenesis, and hampers cell growth [38, 39]. These findings suggest that the roles of vimentin and MMP9 in cancer metastasis may go beyond being mere markers for EMT. Overall, the results of this study shed light on the potential mechanisms underlying the additive inhibitory effect of the combination treatment of sorafenib and 5-MTP on LLC cell migration and metastasis, and emphasize the significance of vimentin and MMP9 as potential therapeutic targets in the context of lung cancer metastasis.

In the present study, sorafenib was found to reduce the expression of phosphorylated STAT3 but increase the expression of phosphorylated Akt in LLC cells. The AKT signaling pathway has been implicated in the development, progression, and metastasis of numerous cancers [39–41]. Therefore, the elevated Akt level induced by sorafenib may influence the efficiency of reducing cancer growth and metastasis. On the other hand, STAT3 is an oncogenic transcription factor known to play a role in various biological functions during human tumor development, including proliferation, survival, and inflammation [42]. It acts as a transcription activator by binding to promoters in a tyrosine phosphorylation-dependent manner, and it is a critical modulator of EMT [43]. In several types of cancer, including lung cancer, STAT3 is constitutively active, leading to continued expression of target genes that promote cell proliferation, survival, and invasion [44]. In the present study, the combination treatment of sorafenib and 5-MTP not only reduced the expression of phosphorylated STAT3 but also decreased the expression of phosphorylated Akt, which may contribute to an additive inhibitory effect compared to that of single treatments. This dual inhibition of both Akt and STAT3 signaling pathways by the combination treatment could be a potential mechanism underlying the enhanced inhibitory effects on cancer growth and metastasis observed in the study.

The LLC mouse model is a widely used model in lung cancer research, including studies on the pathogenesis of lung cancer, drug treatments, and biological therapies [21]. This model is favored due to its ease of production, low cost, and high rate of tumor formation. In our study, we found that the combination treatment of sorafenib and 5-MTP resulted in less tumor growth and metastasis compared to the treatment with sorafenib alone. These results indicate that the combination treatment of sorafenib and 5-MTP may potentially mitigate side effects or drug resistance issues associated with sorafenib treatment, which are important aspects worth investigating further. In conclusion, our findings suggest that 5-MTP enhances the antitumor and anti-metastasis effects of sorafenib by mediating the expression of phosphorylated STAT3 and Akt. These results provide a valuable experimental basis for considering the combination treatment of 5-MTP and sorafenib as a potential therapeutic strategy for lung cancer patients.

### Electronic supplementary material

Below is the link to the electronic supplementary material.

Supplementary Material 1

## Data Availability

The datasets supporting the conclusions of this article are included within the article.

## Abbreviations

5-MTP: 5-methoxytryptophan
LLC: Lewis lung carcinoma
EMT: Epithelial-mesenchymal transition
NSCLC: Non-small cell lung cancer
STAT3: Signal transducer and activator of transcription 3
CI: Combination Index
PBS: Phosphate-buffered saline
IC50: Half maximal inhibitory concentration
CDI: Coefficient of drug interaction
MMP9: Matrix metalloproteinase-9
