# Supplementary material for: 5-Methoxytryptophan enhances the sensitivity of sorafenib on the inhibition of proliferation and metastasis for lung cancer cells
Source: BMC Cancer. 2024 Feb 22;24:248. doi: 10.1186/s12885-024-11986-4 (PMC10885375; doi:10.1186/s12885-024-11986-4)
Supplement: Supplementary file 1 — Supplementary Material 1 [file 12885_2024_11986_MOESM1_ESM.pdf]

Cyclin D1

Cyclin B1

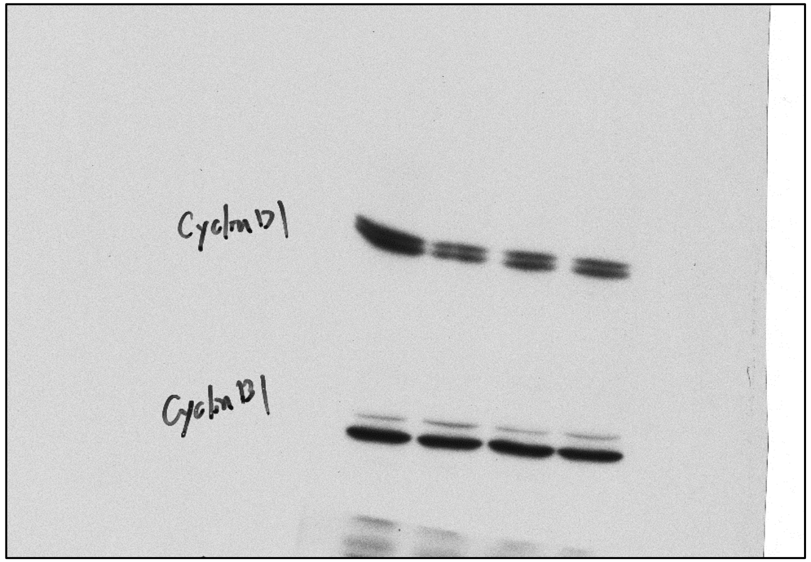

CDK4

CDK1

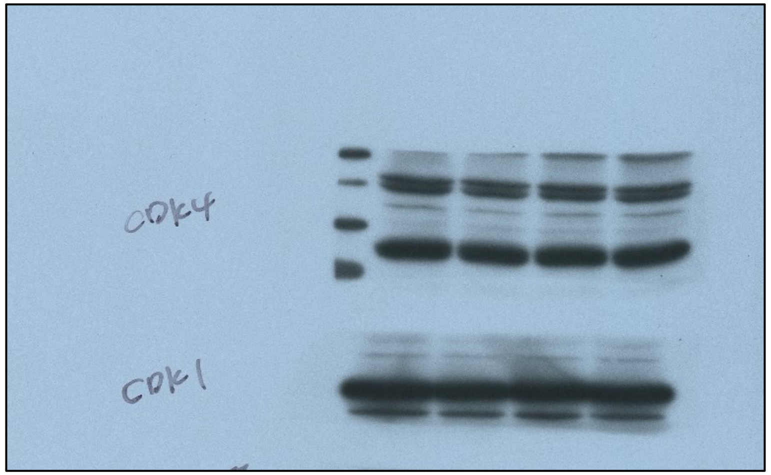

GAPDH

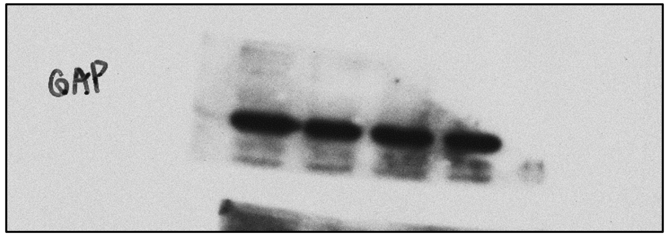

Cyclin D1

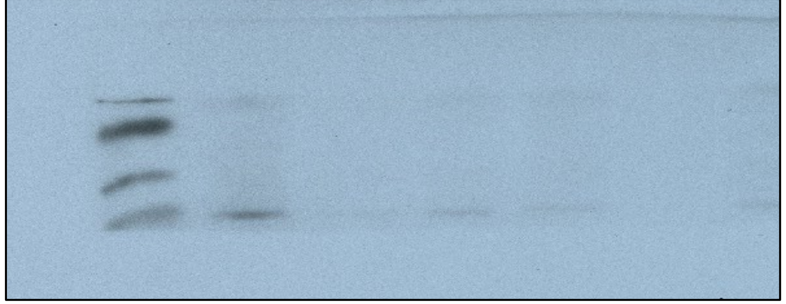

Cyclin B1

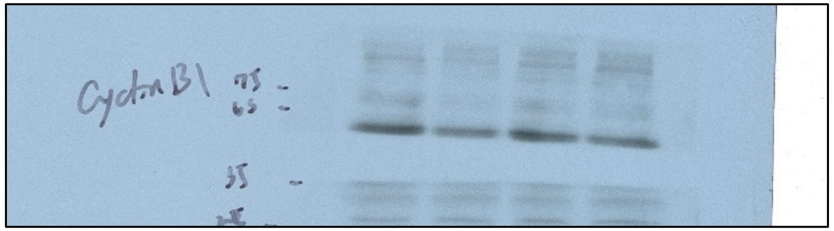

CDK4

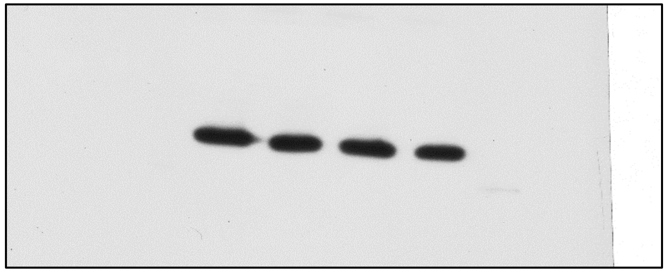

CDK1

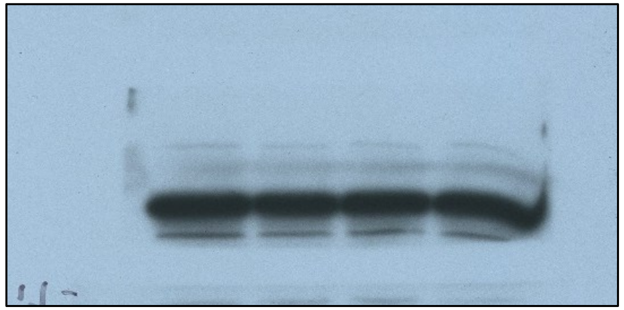

GAPDH

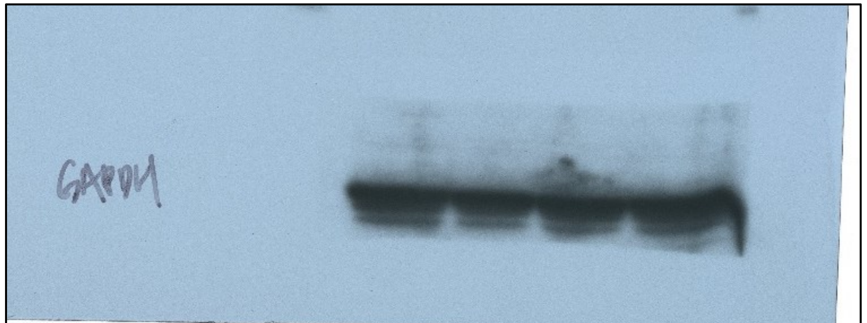

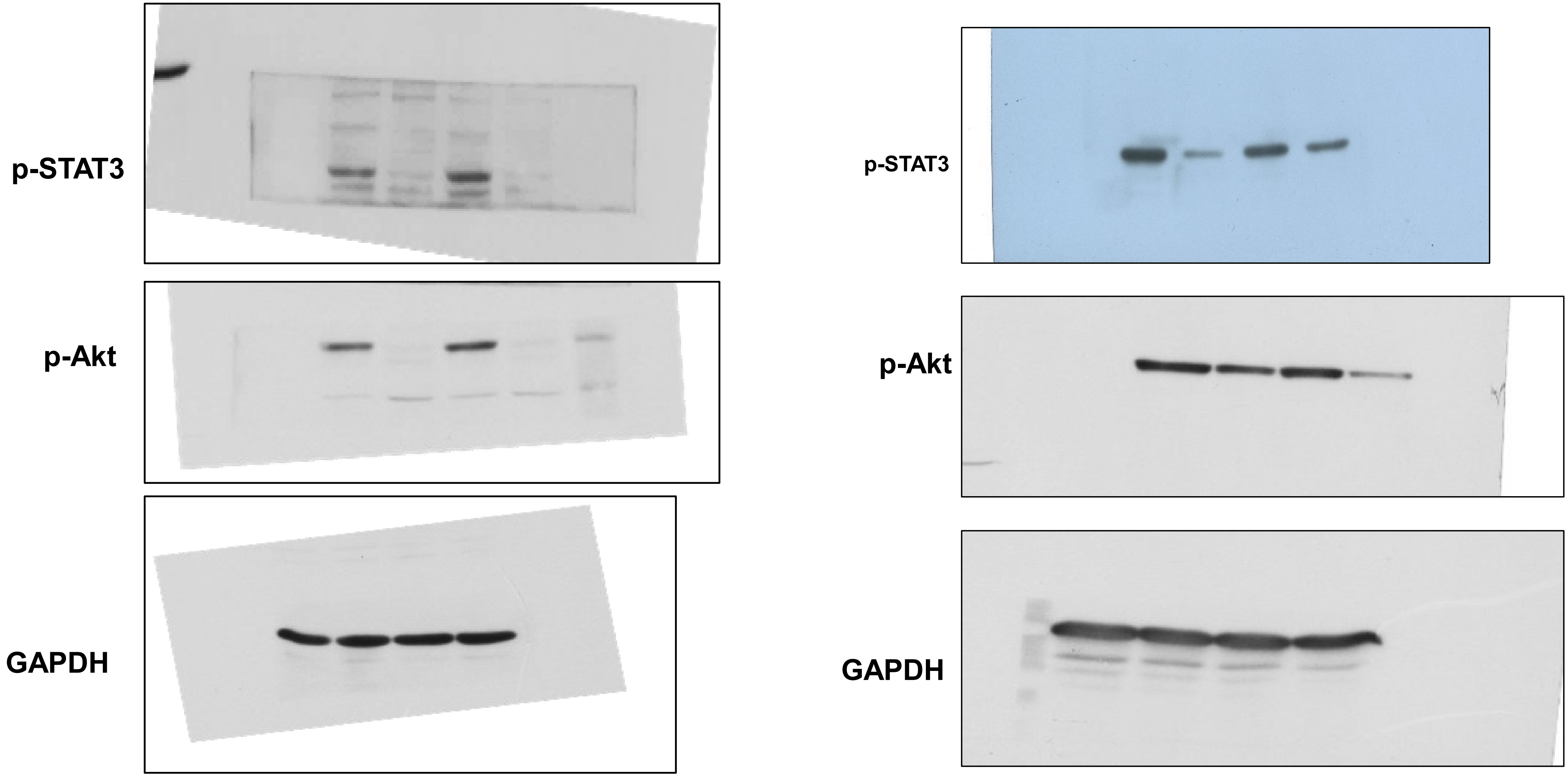
p-STAT3
p-Akt
GAPDH
p-STAT3
p-Akt
GAPDH

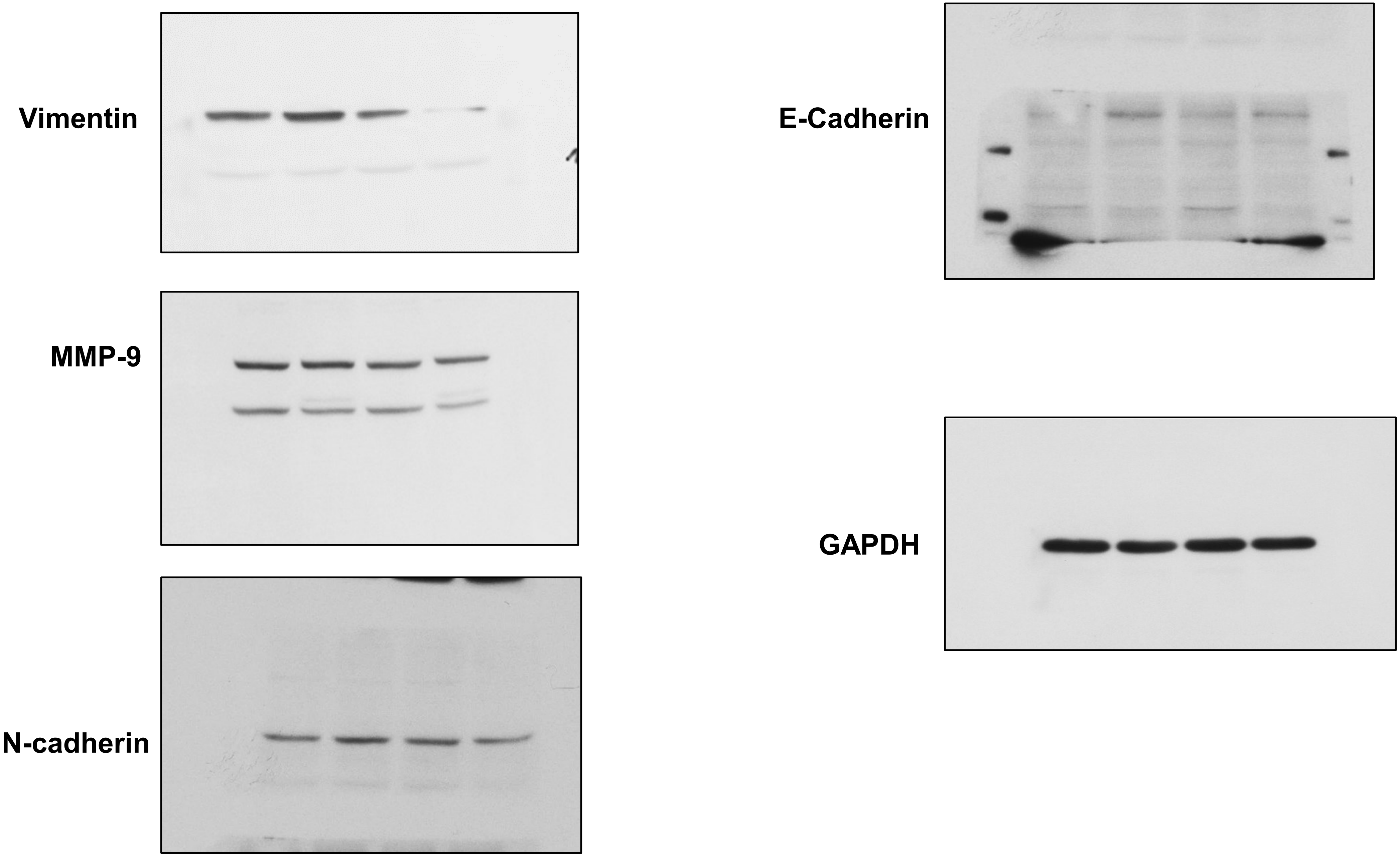
Vimentin
MMP-9
N-cadherin
E-Cadherin
GAPDH

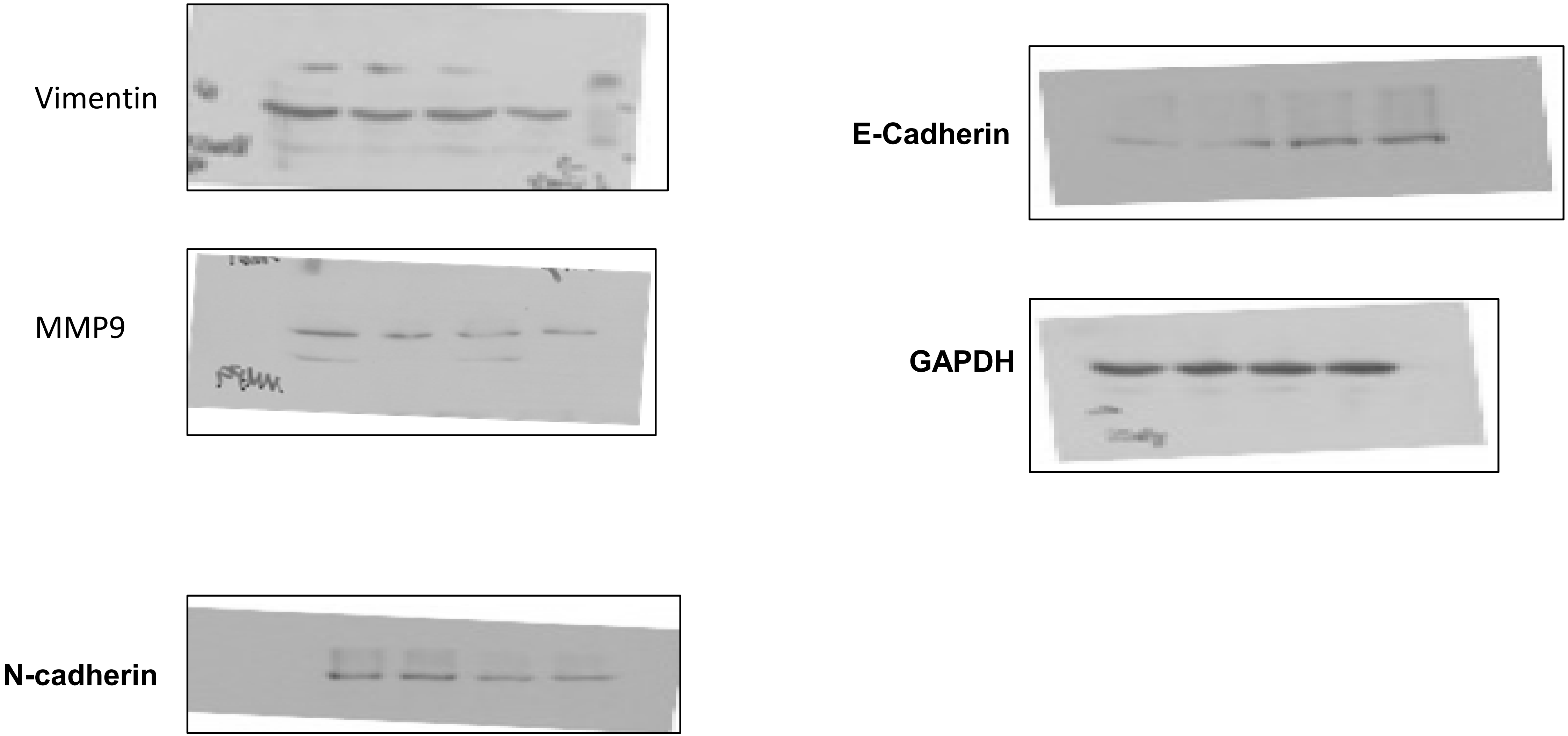
Vimentin
MMP9
N-cadherin
E-Cadherin
GAPDH
